# Genetic polymorphisms in warfarin and tacrolimus-related genes *VKORC1*, *CYP2C9* and *CYP3A5* in the Greek-Cypriot population

**DOI:** 10.1186/1756-0500-7-123

**Published:** 2014-03-05

**Authors:** Despina Hadjipanagi, Stephanie Chrysanthou, Konstantinos Voskarides, Constantinos Deltas

**Affiliations:** 1 Molecular Medicine Research Center and Laboratory of Molecular and Medical Genetics, Department of Biological Sciences, University of Cyprus, Nicosia, Cyprus

**Keywords:** *CYP2C9*, *CYP3A5*, *VKORC1*, Cyprus, Greek-Cypriots, Pharmacogenetics, Population genetics

## Abstract

**Background:**

Two variants in the gene encoding the cytochrome P450 2C9 enzyme (*CYP2C9*) are considered the most significant genetic risk factors associated with bleeding after warfarin prescription. A variant in the vitamin K epoxide reductase (*VKORC1*) has been also associated by several studies with warfarin response. Another variant in the P450 3A5 enzyme (*CYP3A5*) gene is known to affect the metabolism of many drugs, including tacrolimus.

**Findings:**

We conducted a population genetic study in 148 unrelated healthy Greek-Cypriot volunteers (through PCR-RFLP assays), in order to determine the frequencies of the above pharmacogenetics variants and to compare allele frequencies with those in other major ethnic groups. The allele frequencies of CYP2C9*2, CYP2C9*3 and CYP3A5*3 were found to be 0.162, 0.112 and 0.943 respectively, whereas *VKORC1* - 1639A was 0.534. The latter frequency differs significantly when compared with Caucasians, Asians and Africans (p < 0.001) and is still significant when compared with the geographically and culturally closely related to Greek-Cypriots, Hellenes of Greece (p = 0.01). Interestingly ~18% of our population are carriers of four or three risk alleles regarding warfarin sensitivity, therefore they have a high predisposition for bleeding after taking high or even normal warfarin doses.

**Conclusions:**

Our data show no significant difference in the frequency of *CYP2C9* and *CYP3A5* allelic variants when compared to the Caucasian population, but differ significantly when compared with Africans and Asians (p < 0.001). Also, the frequency of variant *VKORC1* - 1639A differs between Greek-Cypriots and every other population we compared. Finally, about 1/5 Greek-Cypriots carry three or four risk alleles and ~50% of them carry at least two independent risk alleles regarding warfarin sensitivity, a potentially high risk for over-anticoagulation.

## Findings

### Background

There are genetic variations in genes encoding for the hepatic CYP450 metabolizing enzymes that can lead to undesirable drug reactions or inadequate response to commonly prescribed doses, either because of reduced or enhanced enzyme activity, respectively [1]. Testing for the activity of the CYP450 enzymes and isoforms before a patient receives a specific medication can provide valuable information concerning the initial dose. Molecular genetic testing is advantageous and more cost-effective compared to enzymatic methods due to methodological simplicity.

It is notable that in August 2007, the FDA label for warfarin, a frequently used anti-coagulant drug, was updated to highlight the benefit of genetic testing to predict warfarin response. This was renewed on February 2010 (http://www.fda.gov/). Candidate-gene association studies have identified two genes responsible for the main proportion of warfarin response: *CYP2C9*, which codes for the enzyme cytochrome CYP450 2C9 that metabolizes S-warfarin [2] and *VKORC1*, which codes for warfarin’s target, vitamin K epoxide reductase [3]. CYP2C9*2 (Arg 144 to Cys) and CYP2C9*3 (Ile 359 to Leu) are recognized as the main *CYP2C9* variants in humans, affecting negatively the enzyme activity [4,5]. In order to avoid high risk for bleeding, these polymorphisms must be taken into account before warfarin treatment [6]. Warfarin-dosing algorithms that incorporate *CYP2C9* and *VKORC1* genetic profile seem to predict pretty accurately the required warfarin dose for each patient [7,8].

The *CYP3A5* gene represents the major extrahepatic isoform of CYP3A gene family and in association with *CYP3A4* are responsible for the metabolism of over 50% of all the clinically used drugs [9]. Some studies pay special attention at the frequent CYP3A5*3 allele and tacrolimus dosing [10], a frequently used immunosuppressant drug. It is recommended that homozygous CYP3A5*3/*3 patients receive roughly a 50% lower initial tacrolimus dose than patients with at least one wild type CYP3A5*1 allele as a result of the difference observed in oral clearance [10,11].

According to the available bibliography, it is more than evident that different human populations can differ significantly in genetic allelic frequencies for drug metabolism genes [12]. The purpose of the present study was to investigate for the first time the prevalence of the most common allelic variants of *VKORC1*, *CYP2C9* and *CYP3A5* in a representative sample of the Greek-Cypriot population, and to compare these data with existing published data from other populations.

### Methods

#### Subjects

Genomic DNA isolated from whole peripheral blood [13] from 148 male healthy unrelated Greek-Cypriot subjects over 20 years old, was used for this study. The population of Greek-Cypriots is 660,000 (census of 2011), therefore the number of 148 individuals represents adequately our population. The subjects were selected according to their origin, aiming at even geographical coverage of all the provinces of Cyprus, including mountainous areas, thereby avoiding population stratification. We enrolled only male subjects for this study due to the fact we had more information about their geographic origin in contrast with females, because of their participation in another phylogenetics project. The DNA samples were used after they were anonymized. Our research project has been approved by the Cyprus National Bioethics Committee. Informed consent was obtained from all subjects. Research carried out on volunteers of this study was in compliance with the Helsinki Declaration.

### Molecular genetic analysis

In this study we directly tested our sample group only for the four following genetic variants: *VKORC1* g.-1639G/A, CYP2C9*2, CYP2C9*3 and CYP3A5*3. We did not test specifically for the wild type CYP2C9*1 allele, the frequency of which was inferred from the absence of the two variants tested. The genetic polymorphisms were genotyped by PCR-RFLP assays. Details of the methods regarding the *VKORC1* g.-1639G/A, CYP2C9*2 and CYP3A5*3 polymorphisms are described elsewhere [14-16]. For the CYP2C9*3 polymorphism, the following primers were designed and used for amplification:

CYP2C9-3_For: 5’ ACACAGATGCTGTGGTGCACGAGGTCCAGAG ***G*** TAC 3’

CYP2C9-3_Rev: 5’ CTGGAAACAAGAGAAAGTCCAGTTAAACTGCCATAC 3’

The forward primer was modified by substitution of the fourth nucleotide from the 3’ end, from A to G (italics and bold), so that upon amplification a restriction recognition site for *BanI* is created. The digestion products were analyzed by electrophoresis on 3.5% agarose gels. The CYP2C9*3 allele is represented by the 225 bp and 31 bp bands, whereas wild type allele is represented by the uncut 256 bp band.

#### Statistical analysis

Comparison of population allele frequencies was performed through Pearson chi-square test, using SPSSv.15 statistical package (IBM, USA). The significance level, alpha, was set to 0.05. Hardy-Weinberg equilibrium was tested for all the analyzed polymorphisms through an Excel based application (Microsoft Office 2007).

### Results and discussion

Genotypic frequencies for the tested pharmacogenetics variants in our population are tabulated in Table 1. The genotypic frequencies for each variant are in Hardy-Weinberg equilibrium. All the tested Greek-Cypriots are carriers of the CYP3A5*3 allele (no homozygous wild-type CYP3A5*1/*1 were found), which is responsible for reduced tacrolimus clearance, while 88.5% of them are homozygous CYP3A5*3/*3 (Table 1). These frequencies are very similar with those for the Hellenic population in Greece and generally with those for the Caucasian population [12,17]. We paid special attention at genotypic combinations for the three genotyped warfarin related genes (Table 2). We believe that clinicians in Cyprus have to take seriously into account that ~18% of the Greek-Cypriot population they serve are carriers of four or three risk alleles and about 50% carry at least two risk alleles, predisposing for bleeding after taking high warfarin doses. The combinatorial frequency of *CYP2C9* and *VKORC1* genotypes in the target population appears higher than expected based on individual genotypes, but we do not have a convincing explanation for this. Individuals carrying six or five risk alleles were not detected. Interestingly, we found that 2.7% of subjects in our population sample are simultaneously carriers for the three tested variants (Table 2), a frequency that is exactly the same with what is reported in Jewish populations [18].

**Table 1:** Genotype distribution of the four pharmacogenetics variants in the Greek-Cypriot population

	***VKORC1 *-1639AG**			***CYP2C9***			***CYP2C9***			***CYP3A5***		
**n**	**AA**	**AG**	**GG**	*1/*1	*1/*2	*2/*2	*1/*1	*1/*3	*3/*3	*1/*1	*1/*3	*3/*3
148	44	70	34	107	34	7	115	33	0	0	17	131
100	29.7%	47.3%	23.0%	72.3%	23.0%	4.7%	77.7%	22.3%	0%	0%	11.5%	88.5%

**Table 2:** Genotypic combinations of the three genotyped variants related with warfarin, in 148 Greek-Cypriot individuals

**Genotypic combinations**			**No**	**%**
*VKORC1* -AA	CYP2C9*2/*2	CYP2C9*1/*3	0	0
*VKORC1* -AA	CYP2C9*2/*2	CYP2C9*1/*1	2****	1.35
*VKORC1* -AA	CYP2C9*1/*2	CYP2C9*1/*3	2****	1.35
*VKORC1* -AG	CYP2C9*1/*2	CYP2C9*1/*3	2***	1.35
*VKORC1* -AG	CYP2C9*2/*2	CYP2C9*1/*1	3***	2.03
*VKORC1* -AA	CYP2C9*1/*2	CYP2C9*1/*1	7***	4.73
*VKORC1* -AA	CYP2C9*1/*1	CYP2C9*1/*3	10***	6.76
*VKORC1* -AG + GG	CYP2C9*1/*1	CYP2C9*1/*3	19	12.84
*VKORC1* -AA	CYP2C9*1/*1	CYP2C9*1/*1	23	15.54
*VKORC1* -AG + GG	CYP2C9*1/*2 + *2/*2	CYP2C9*1/*1	25	16.89
*VKORC1* -AG + GG	CYP2C9*1/*1	CYP2C9*1/*1	55	37.16
SUM			148	100

No CYP2C9*3/*3 genotypes were observed.

****Individuals with four risk alleles (2.70%).

***Individuals with three risk alleles (14.87%).

It has been proven that CYP2C9*2 and *3 cause a reduction in S-warfarin clearance (10-fold variation observed from CYP2C9*11 to CYP2C9*33) activity (*1/*1 > *1/*2 > *1/*3 > *2/*2 > *2/*3 > *3/*3). The effect of the CYP2C9*3/*3 genotype is the most severe one with clearance of S-warfarin being 10% of the wild type genotype [19]. *VKORC1* encoded enzyme interacts with warfarin, so the enzyme levels can affect sensitivity to warfarin. Jorgensen et al. [20], reviewed recently almost all the available bibliography and found that CYP2C9*3 and *VKORC1* variants are significant for warfarin dose determination for most ethnic populations [20]. Sconce et al. [7] showed clearly (giving also a useful algorithm for warfarin dose determination) that the three significant variants of the aforementioned genes have additive properties [7].

In conclusion, it is evident through population specific studies, like the one we present here, that pharmacogenetics allele frequencies can differ significantly in different sub-populations. The results of our study compare well to those of other Caucasian populations, but with some notable differences, like the increased frequency of the *VKORC1-*1639A allele (Table 3). Allele frequencies between the six provinces of Cyprus show high similarity (Table 3). We must have in mind that the Greek-Cypriot population used to be isolated and small, and therefore susceptible to migratory waves and genetic drift phenomena. Even comparing with the Greek population of Greece, that we are closely related (geographically and culturally), there are still some significant differences (Table 3). This fact underlines the necessity for population-specific allelic frequency studies, since these determinations can be helpful for the prescribing clinicians and their patients.

**Table 3:** Allele frequencies of the four studied pharmacogenetic variants in Cyprus and comparison with other populations

**Population group**	**No of tested chromosomes**	***VKORC1 *-1639A**	***CYP2C9*** *2	***CYP2C9*** *3	***CYP3A5*** *3	**Reference**
**Greek-Cypriots**	**296**	**53.38%**	**16.20%**	**11.15%**	**94.26%**	This study
Nicosia	106	62.26%	18.87%	9.43%	95.28%	
Limassol	52	44.23%	19.23%	11.54%	96.15%	
Ammochostos	60	51.67%	15.00%	15.00%	93.33%	
Larnaka	28	50.00%	14.29%	7.14%	92.86%	
Pafos	32	50.00%	9.38%	9.38%	90.63%	
Kerynia	18	44.44%	11.11%	16.67%	94.44%	
Caucasians	1362	37.81%				[21]
		(P < 0.001)				
	3484		14.00%	6.40%		[12]
			(P = 0.296)	(P = 0.002)		
	284				95.50%	[12]
					(P = 0.526)	
Africans	736	10.05%				[21]
		(P < 0.001)				
	1376		2.20%	1.80%		[12]
			(P < 0.0001)	(P < 0.001)		
	1330				31.80%	[12]
					(P < 0.001)	
Eastern Asians	1282	91.03%				[21]
		(P < 0.001)				
	13284		<0.1%	3.30%		[12]
			(P < 0.001)	(P < 0.001)		
	7658				76.10%	[12]
					(P < 0.001)	
Hellenes from Greece	656	44.40%				[22]
		(P = 0.010)				
	566		12.90%	8.13%	94.35%	[17]
			(P = 0.183)	(P = 0.144)	(P = 0.957)	

In parenthesis: p-value after comparison with the Greek-Cypriot frequencies.

## Competing interests

The authors declare that they have no competing interests.

## Authors’ contributions

CD and KV conceptualized the study, collected the samples, supervised genotyping and wrote the manuscript. KV performed the statistical analysis. DH performed most of the genotyping of this study and she categorized the data. SC genotyped the *VKORC1* variant. All authors read and approved the final manuscript.
